# Lessons Learned in Change Management in Deploying Novel Informatics Solutions: Experience Implementing a Point-of-Care Patient Photography System with Radiography

**DOI:** 10.1007/s10278-023-00796-y

**Published:** 2023-06-15

**Authors:** Carson A. Wick, Srini Tridandapani, Marta E. Heilbrun, Tarek Hanna, Nabile Safdar, Pamela Bhatti

**Affiliations:** 1 Camerad Technologies, LLC, Decatur, GA 30033 USA; 2 School of Electrical and Computer Engineering, Georgia Institute of Technology, Atlanta, GA 30308 USA; 3 Department of Radiology and Imaging Sciences, Emory University, Atlanta, GA 30332 USA; 4 Department of Radiology, University of Alabama at Birmingham, 619 19th Street South, JT N455E, Birmingham, AL 35249-6830 USA; 5 Imaging Services, Intermountain Healthcare, Salt Lake City, UT 84111 USA

## Abstract

We describe implementation of a point-of-care system for simultaneous acquisition of patient photographs along with portable radiographs at a large academic hospital. During the implementation process, we observed several technical challenges in the areas of (1) hardware—automatic triggering for photograph acquisition, camera hardware enclosure, networking, and system server hardware and (2) software—post-processing of photographs. Additionally, we also faced cultural challenges involving workflow issues, communication with technologists and users, and system maintenance. We describe our solutions to address these challenges. We anticipate that these experiences will provide useful insights into deploying and iterating new technologies in imaging informatics.

## Background

A system for automatically acquiring point-of-care patient photographs for portable radiography studies was deployed at Emory University Hospital (EUH), Atlanta, GA, USA. The system seamlessly adds these photographs to corresponding radiology studies, where they are available to all authorized parties.

Intended to provide a visual representation of the patient and their physical environment, these photographs augment the information contained in the standard radiology images [1, 2]. Observed benefits of point-of-care photographs include a reduction in patient-misidentification errors [2], reduction in laterality errors [3], pre-exam planning by technologists, increased radiologist confidence [4], and accelerated reconciliation times. Moreover, photographs may be used retrospectively for quality assurance and improvement measures with the potential for medicolegal applications [1, 2].

This paper is intended to share both our technical and cultural experiences in deploying an imaging informatics innovation. We anticipate that it will provide useful insights into deploying and iterating new technologies, particularly for radiology departments, healthcare informatics teams, and small businesses in the radiology space.

### Technical Background

**Fig. 1:**
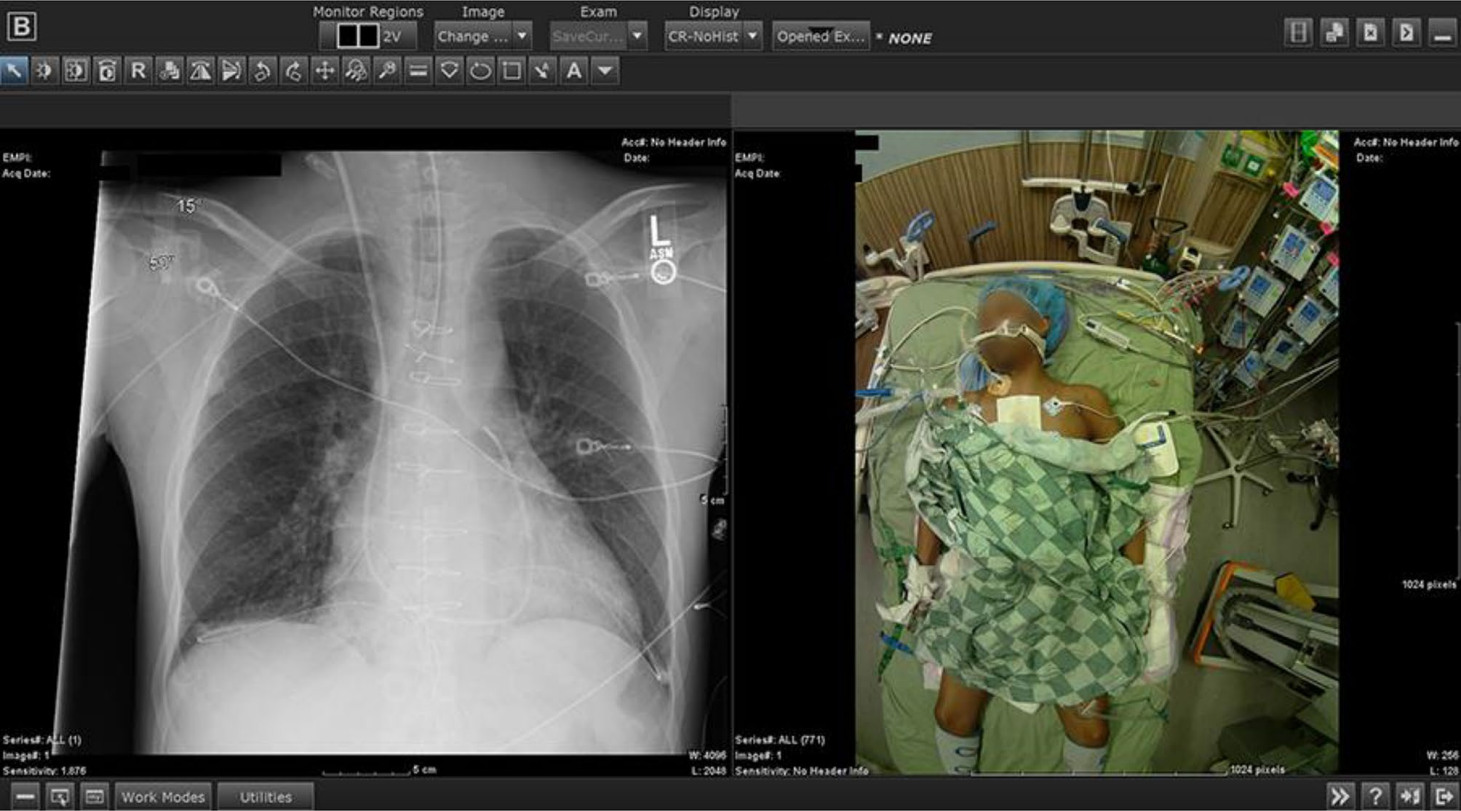
Portable radiograph along with the simultaneously acquired point-of-care photograph displayed at a PACS workstation

**Fig. 2:**
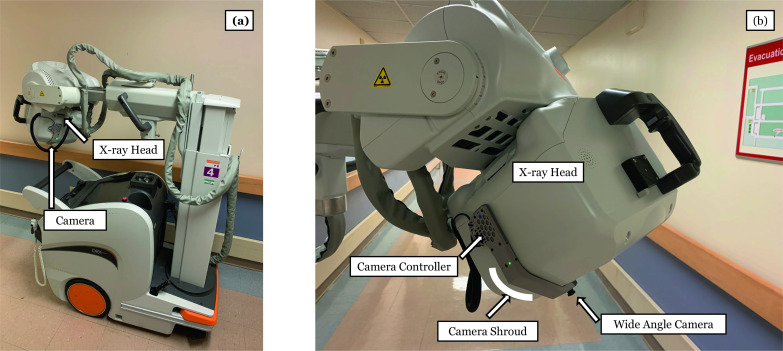
**a** Point-of-care camera attached externally to a portable radiography machine. **b** Close-up view of the camera, camera controller, and camera shroud on the X-ray head of a portable radiography machine

The point-of-care photography system produces patient photographs that are archived with simultaneously obtained digital radiograph images (Fig. 1). The system consists of two main components: (1) miniature programmable cameras to acquire photographs (Fig. 2) and (2) a system server to retrieve, process, and integrate these photographs within the hospital information systems environment, which for the initial implementation corresponded to the picture archiving and communications system (PACS).

#### Programmable Camera

Custom programmable cameras are attached to portable radiography machines (hosts) without requiring any modification to the host machine. The programmable cameras consist of a wide-angle camera (SainSmart, Lenexa, KS, USA) and a micro-computer (Raspberry Pi Foundation, Cambridge, England, UK) running a Linux-based operating system. The cameras communicate with the system server wirelessly and are powered by an available USB port on the rear of the host machine. All sensitive data, namely photographs, is deleted upon retrieval by the system server. Prior to retrieval, temporary photograph data is stored on an encrypted volume that can only be unlocked by the system server. Furthermore, the cameras have no access to the internet, and communication on the network is restricted to only the system server. As devices not managed by the institution, the cameras are routinely updated by the vendor to address any emergent security software vulnerabilities. Photograph acquisition is triggered automatically and concurrently with X-ray exposure.

#### System Server

The system server is installed onsite at EUH in a secure environment. The server is a general-purpose, Linux-based computer and performs all necessary steps to automatically deliver a photograph series to the correct radiography study. These steps include (1) retrieval and deletion of photographs from cameras, (2) retrieval of radiograph metadata, (3) matching of photographs and radiographs, (4) processing photographs, (5) converting photographs to DICOM format with information from matching radiograph, (6) sending photograph to PACS, and (7) archiving and/or deleting image data as appropriate [5, 6].

### Cultural Background

The point-of-care patient photography system was deployed at EUH, a hospital performing roughly 5000 portable radiography studies per month in the emergency room, intensive care units, and additional in-patient settings. Approximately 60 attending radiologists, 60 radiology residents, and 25 radiography technologists and numerous referring clinicians regularly view these patient photographs. Additionally, any authorized party can review these photographs should the need arise, e.g., confirmation of patient identity or condition.

## Methods

Throughout the deployment and operation of the point-of-care photography system, a number of technical and cultural experiences resulted in system improvements. In this section, we present a description of the original state of the system when first deployed, followed by the related experiences, and lastly the corresponding actions carried out as a result.

## Technical Experiences

We define technical experiences as those that led to changes in the technical make-up of the camera system, i.e., the programmable camera and system server.

### Programmable Camera

#### Wireless Communications

*Challenge 1*: When originally deployed, the programmable camera communicated with the system server via an ad hoc wireless network hosted by the system server. This presented a challenge for multiple reasons. (1) Delays: The system server could only communicate with the programmable cameras when both were within 30 feet (10 m) of each other. As a result, photos could not be retrieved and processed until the portable radiography unit returned to the technologist workroom where the system server resided. Because the radiographs were sent to the PACS wirelessly, this delay could cause photographs to not arrive in PACS in time for radiologist interpretation. (2) Connections: The system monitoring and performance relied on stable and consistent communication between the programmable cameras and the system server. (3) Security: While not an issue in our implementation, we were sensitive to the fact that many medical institutions, e.g., government hospitals, would not have allowed third-party wireless networks on their site, posing a potential limitation to implementation.

*Solution 1*: Working with the hospital, approval was obtained for the programmable cameras to connect to the system server directly using the hospital’s wireless network. For security reasons, the programmable cameras were restricted to communication with only the system server. This direct connection solved the issues related to delay, persistent connection, and security.

#### Automatic Photograph Acquisition

**Fig. 3:**
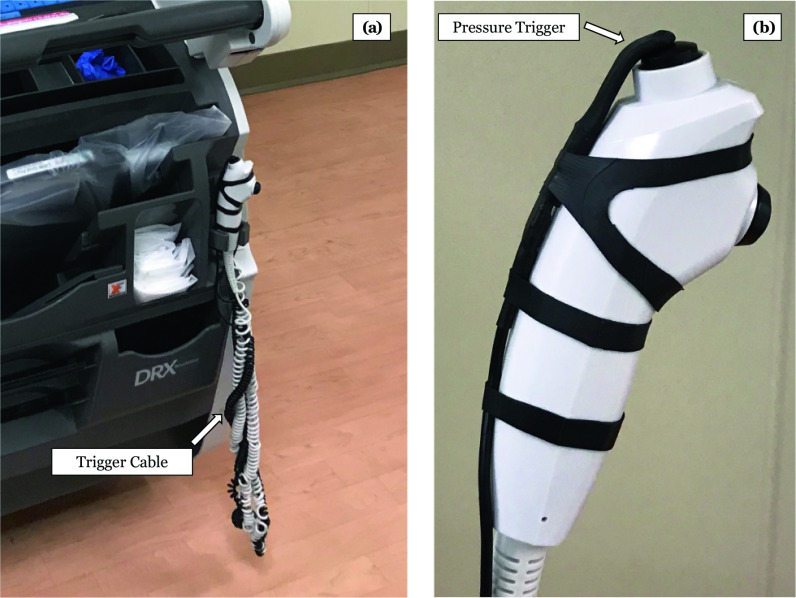
**a** Initial hardware implementation for simultaneous triggering of photography acquisition. The pressure sensor is attached to the portable radiography machine trigger. **b** The camera trigger cable is strung along the radiography trigger cable showing how this can be tangled

*Challenge 2*: When originally deployed, camera acquisition was triggered using a pressure sensor attached to the X-ray machine handswitch. The pressure sensor would also be pressed and activated when the technologist pressed the handswitch button. The sensor was suspended over the handswitch button on a piece of semi-rigid nylon and covered in a latex membrane to prevent liquid ingress and contamination (Fig. 3a). A long cord connected the pressure sensor to the programmable camera on the X-ray machine head (Fig. 3b).

Throughout the initial phases of the deployment, it became clear that this approach was suboptimal in the following ways. (1) Fragility: The pressure switch could be dislodged so that it was no longer pressed when the handswitch trigger was pressed. The latex membrane developed fissures and broke down after 1 month of use. (2) Manipulation: Some technologists were deliberately bypassing triggering photograph acquisition by moving the pressure switch to the side. (3) Clutter: The coiled cable of the pressure sensor would become tangled with the coiled cable of the handswitch.

**Fig. 4:**
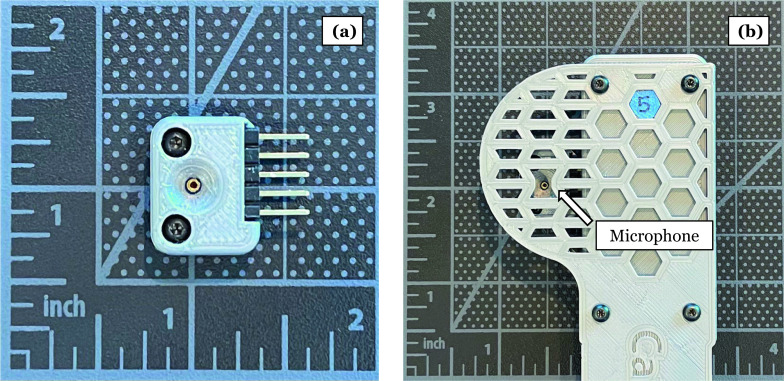
A microphone (**a**) was embedded along with the on-board camera controller (**b**) to trigger the camera when the audible alert from the radiography machine was emitted during radiograph acquisition

*Solution 2*: To streamline the system by reducing the number of components, the pressure sensor trigger and the associated cabling were replaced with an auditory-based trigger [7]. A microphone was added to the programmable camera and software developed to automatically detect and trigger photograph acquisition based on the auditory warning tone generated by the radiography machine during X-ray exposure (Fig. 4). Interventions and education with the technologists to the rationale and operation of the system improved acceptance, but did not completely eliminate the deliberate system bypass.

#### Programmable Camera Enclosure

**Fig. 5:**
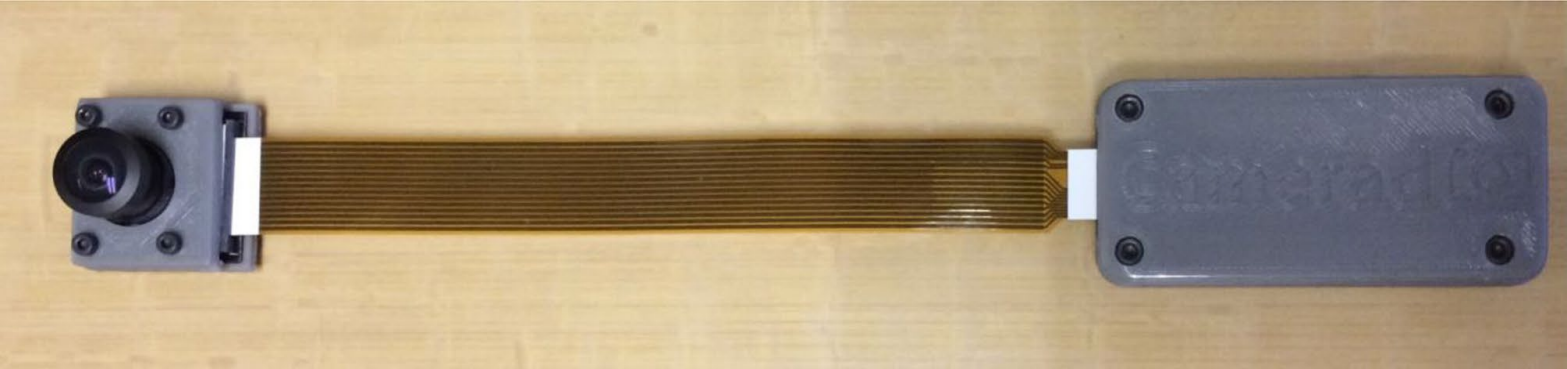
Camera attached to the camera controller with a cable. All three components were housed in the shroud shown in Fig. 2

*Challenge 3*: The programmable camera consisted of a separate enclosure for the camera and camera controller (micro-computer) as shown in Fig. 5. These two devices were connected by a ribbon cable and affixed to the head of the X-ray machine head using adhesive-backed Velcro. It was easy to detach the cameras and controllers and the ribbon cable because technologists often place their hands on the X-ray machine head during the process of radiograph acquisition.

*Solution 3*: A one-piece enclosure was designed and 3-D printed to match the contour of the X-ray machine head, providing more surface area and stability of the programmable camera while protecting the delicate ribbon cable connections (Fig. 2b). Attachment points for securing power and trigger cables were incorporated into the design, and overall, these modifications improved protection of the programmable camera unit.

### System Server

#### System Server Hardware

*Challenge 4*: The camera system was originally deployed using a Raspberry Pi 3 Model B. This choice was made due to the small form factor, accessible pricing, Linux develop environment, and extensive documentation. During the initial stages of development, the computational and storage limitations motivated transitioning to more powerful system server hardware. This enabled photograph auto-rotation capabilities to ensure that the photographs were correctly oriented during interpretation by the radiologist. In brief, auto-rotation performed by the camera system server requires more processing power than the originally deployed hardware. Additionally, the file I/O speeds of the Raspberry Pi were insufficient for archive and backup procedures. Moreover, routinely deploying security patches and software updates to this server proved cumbersome.

*Solution 4*: To overcome the constraints of the Raspberry Pi platform, the system server was ported to a mini personal computer (Intel NUC, Intel, Santa Clara, CA, USA). The dimensions of the new computer (4.6 × 4.4 × 2 inches) allowed for it to be placed securely in the technologist workroom without dedicated space. The computer features an antitheft key lock hole that secures the computer in place. We are currently migrating to a virtual machine on the institutional server system to be managed by the institution. This allows the institution to apply software security updates on the institutions as needed according to their internal policies.

#### Image Processing of Photographs

*Challenge 5*: When first deployed, the goal of the camera system was the automatic and immediate integration of point-of-care patient photographs to the corresponding radiography images. However, it was evident that the photographic image content required processing. Notably, the raw photographs sent to PACS were often too dark due to either dark hospital rooms or the auto-exposure being affected by the bright exposure indicator light from the radiography machine. Additionally, photographs were often displayed incorrectly oriented (e.g., head to the left or right or upside down) due to the lack of rotation information on the raw photographs.

*Solution 5*: An automatic gamma adjustment algorithm [8, 9] that nonlinearly brightens dark image regions more than light image regions rectified the problem of overly dark regions by effectively boosting the dark shadows of the patient photograph (Fig. 7). Because original pixel values are modified by the gamma adjustment, both the original and processed photographs were initially sent to PACS. However, radiologists noted the burden of receiving two of every photograph (original and processed), and the imaging department leadership acknowledged that it was acceptable to send only the processed photograph to PACS. Original photographs are archived and backed up onsite.

Image auto-rotation was accomplished using convolution neural network (CNN) face detector on each of the four possible 90-degree rotations [10, 11]. The rotation with the detected face was deemed the correct rotation. For images with multiple detected faces and/or rotations, the face detected with the strongest response was assumed to be correct.

### Cultural Experiences

Cultural experiences in deploying the camera system led to improvements in communication, hospital workflow, and system support.

#### Communication with Hospital Staff and Radiography Machine Service Personnel

*Challenge 6*: Initially, communication about the project to the relevant stakeholders was primarily performed in person and focused on technical personnel in information systems and radiography technologists. Upon deployment progression, it became clear that all parties supporting the clinical setting were not fully informed about the operation and rationale for deploying the technology. For example, hospital staff as well as vendor service personnel for the radiography machines that were equipped with cameras expressed confusion and concerns, both of which should be minimized before deploying a new technology.

A few radiologists and radiography technologists came forward with appreciable concerns and feedback about the deployment.

Radiologists were concerned with the display of the photographs. First their feedback helped guide the image processing that now occurs on the system server prior to the photograph being sent to PACS. Second, an important concern was the vulnerable state of the patient in some of the photographs. This is discussed in detail below where experiences regarding hospital workflow are given.

Similarly, technologists’ concerns were focused on the privacy and dignity of the patients. In addition, they were uneasy about the possibility the system was being used to monitor the technologists. These concerns led to some technologists preventing automatic photograph acquisition, either by bypassing the original pressure sensor trigger or unplugging the camera power cable from the back of the radiography machines.

*Solution 6*: To address these concerns, a training and question and answer session was created between the camera vendor and the concerned technologists. In addition, literature co-drafted by the camera vendor and radiology leadership providing a brief overview of the rationale and implementation of the technology was distributed electronically to radiology staff. Lastly, the camera vendor’s contact information was posted in the radiography technologist workrooms in case a need for technical support or questions arose. Members of the initial development team met with the Chief Quality Officer and the Chief Medical Officer of the institution to ensure alignment of the initiative at all levels.

When initially deployed, the technology was met with some confusion by the field service personnel from the radiography machine manufacturer. The camera vendor had received permission from both the hospital and the manufacturer. However, this was not communicated to the service personnel that support the radiography machines who were concerned about the attached camera and how it operated. This required additional communication between the camera technology vendor and the regional service manager of the radiography machine vendor. In addition, the consent of the radiography machine manufacturer was formalized for future deployments from machines from that particular manufacturer.

#### Hospital Workflow

*Challenge 7*: During the initial stages of the deployment, radiologists and clinicians expressed concerns regarding the vulnerable state of some patients when radiographed, such as intubated in the ICU, which became apparent because of the accompanying photographs. These concerns were addressed by performing staff training regarding maintaining patient dignity by appropriately draping patients during radiography, decreasing the automatic logout time for hospital workstations that were employed to view patient images, and confirming presence of privacy screens for hospital workstations.

*Solution 7*: A similar concern was expressed by technologists regarding famous or otherwise notable patients and if photographs should be taken in this situation. Working together the camera vendor and hospital, a protocol was developed to remove photographs should they be deemed inappropriate. Presently, this is not a criterion for rejecting photographs. Finally, an override button was also provided on the camera controller to prevent acquisition of a photograph when the technologist deemed that such acquisition was inappropriate.

Prior to deployment, technologists and radiologists had no method to verify the identity of or visualize the patient after the exam took place. Technologists now use the accompanying point-of-care photographs to verify the patient’s identity prior to completing the study by comparing the current photograph to older photographs of the same patient. Photographs are also used in this manner by radiologists during interpretation to verify identity as well as obtain clinical context regarding the patient’s condition. Lastly, prior photographs are now used for pre-exam planning, e.g., large patients that may need an extra technologist to assist.

#### Camera System Support

*Challenge 8*: The support necessary to maintain the point-of-care photography system is minimal and easily performed by hospital staff in most cases. When initially deployed, the camera vendor was responsible for all support tasks. These included replacing worn pressure sensor triggers, reconnecting power cables when unplugged, reattaching cameras to the radiography machine head using Velcro, and correcting the system clock of the radiography machines as it drifts over time. The audio-based trigger eliminated the need to replace the pressure sensor triggers.

*Solution 8*: Throughout the deployment, a number of technologists have come forward in support of the system. These technologists have become super users of the system and are appreciated as a point of contact for the simple support tasks listed above, i.e., reconnecting cameras and updating radiography machine clocks when needed. Additionally, they communicate with the camera vendor regarding any issues with the camera system. Lastly, the camera vendor has provided the technologists with a basic maintenance kit needed to perform these tasks.

## Results

### Technical

Technical experiences throughout deployment led to substantial improvements in both the programmable camera and server of the point-of-care photography system.

#### Programmable Camera

**Fig. 6:**
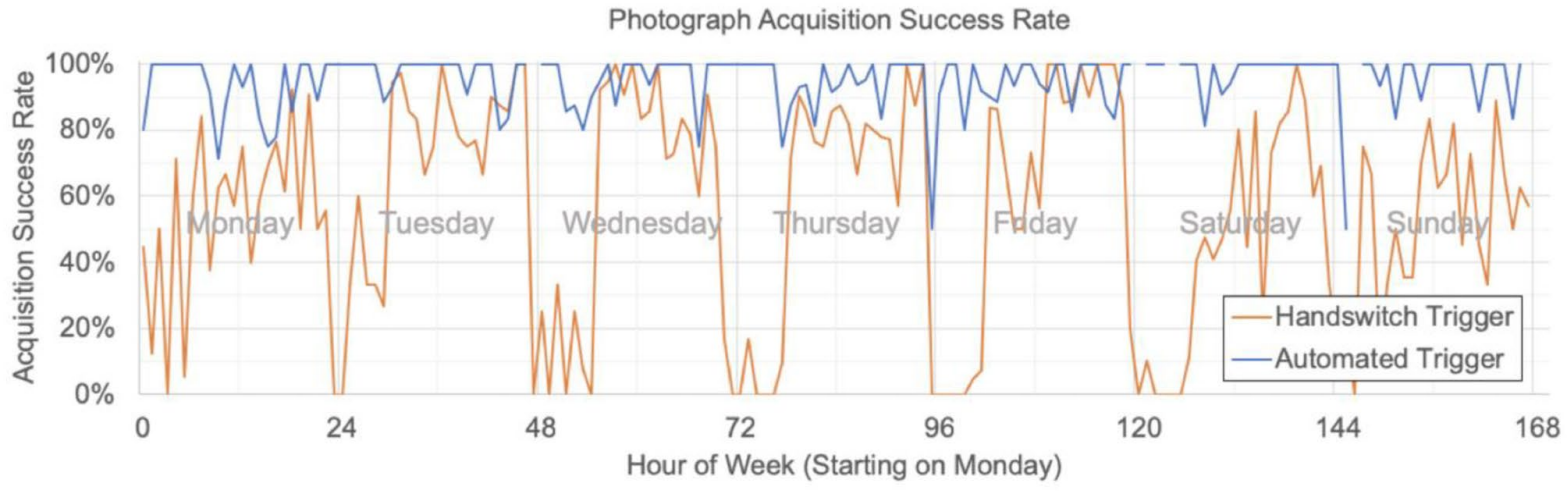
Photograph acquisition success rate increased and was more consistent when automated audible trigger was implemented over the prior handswitch trigger

Three main modifications improved the utility of the programmable camera. First, the audio-based automatic triggering increased the reliability of photograph acquisition and reduced intentional bypassing of triggering. The acquisition success rate using both the original pressure-based and the audio-based trigger is shown as a function of hour of the week in Fig. 6. At baseline with the original trigger, the median and average hourly success rates were 66.7% and 56.3%, respectively. After implementing the audio-based trigger, the corresponding rates were both higher and more consistent, with a median and average of 100% and 95.3%, respectively. Second, the updated wireless approach, whereby each camera is connected to the hospital wireless network, led to a persistent connection between each camera and the server, minimizing delay and enhancing system status monitoring. Third, the unified enclosure of the programmable camera eliminated the problem of disconnected and damaged camera cables and minimized the frequency with which the camera was dislodged from the radiography machine head.

#### System Server

**Fig. 7:**
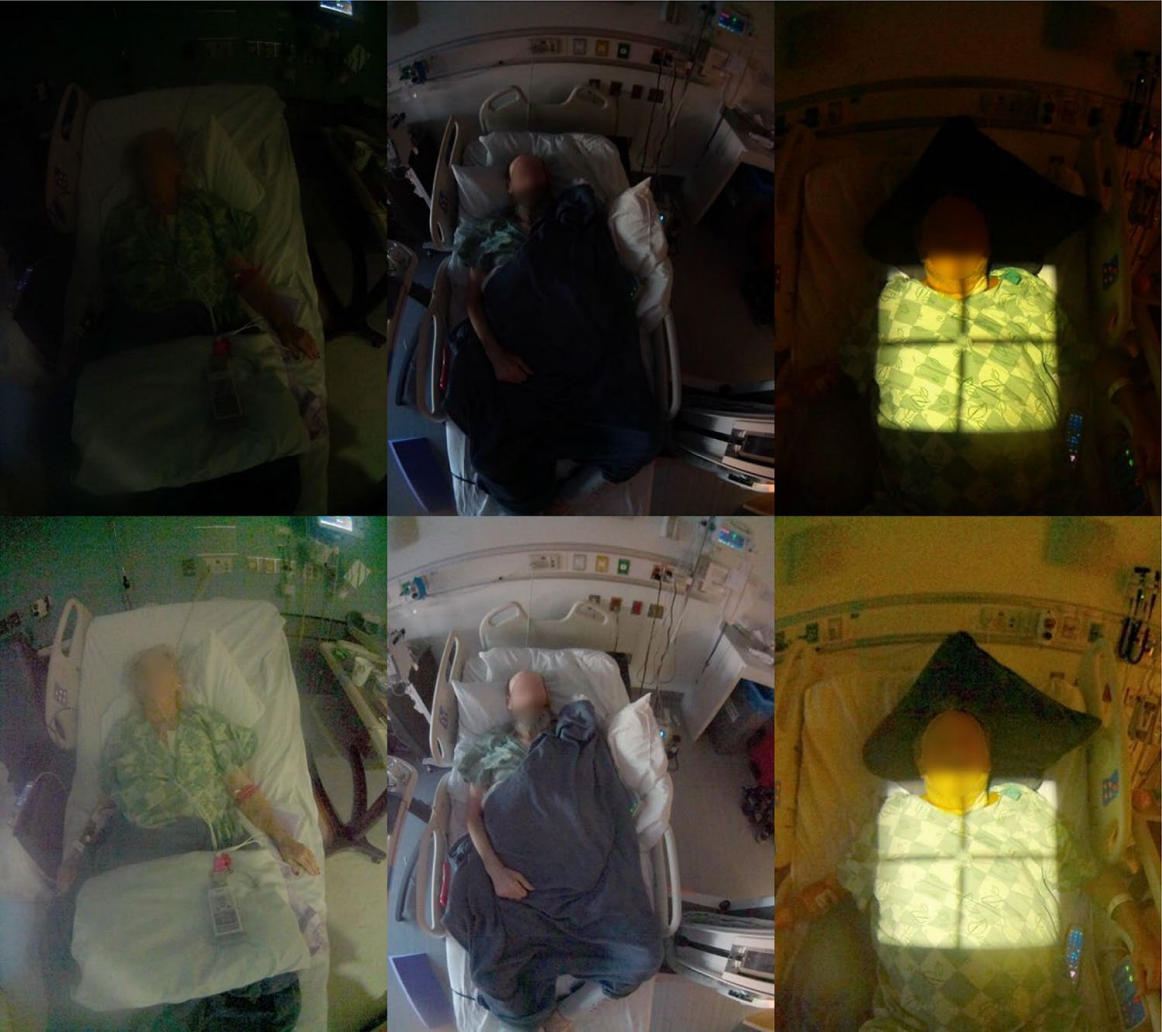
Examples of automated gamma correction to reveal dark image regions. Original images are above, and corresponding, corrected images are below. Patient faces are blurred for privacy in publication but not in practice

The updated hardware used for the system server decreased the per-photo processing time to approximately 20 s from over 2 min. The majority of this processing time is spent performing the CNN face detection used for photograph auto-rotation which for this initial deployment was correct for approximately 73% of non-extremity photographs. After implementing automatic gamma adjustment, patient faces and their surroundings can be visualized in all but the darkest of rooms. An example of the pre- and post-gamma adjusted images is shown in Fig. 7. Lastly, by only sending the processed photo to PACS, a reduction of 35% in the total number of photographs sent to PACS was achieved. This number is less than 50% because not all photos required gamma-adjustment, i.e., they are bright enough that the original image is sent.

Additionally, to decrease the burden with routine manual security updates to the server, we are migrating to a virtual server system, wherein the security updates can be managed automatically by the institution’s information technology division.

### Cultural

Importantly, cultural experiences throughout deployment led to improvements in communication, hospital workflow, and system support.

### Communication

The communication and understanding about the deployment between the camera vendor, hospital leadership, and hospital staff has improved greatly. For some hospital staff, it was helpful and reassuring to hear that the top leaders were supportive of the changes. The experiences throughout deployment provided on opportunity to build a network between the stakeholders. Additionally, there are now channels for future concerns to be brought up and addressed going forward.

**Fig. 8:**
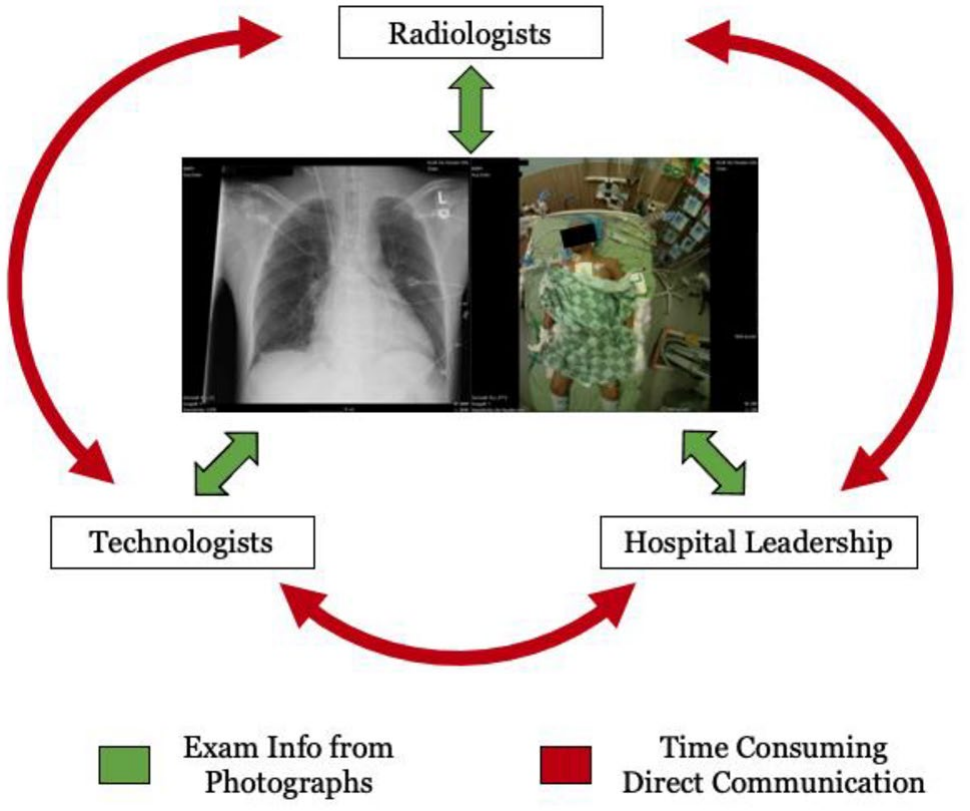
The photographs obtained along with the radiographs provided communication between various stakeholders in the hospital. For example, technologists could communicate with radiologists about patient conditions and the need for non-standard positions. Hospital leadership and radiologists could see conditions in the hospital rooms needing changes to standard operating procedures

Prior to the implementation of the patient photography system, there was no method for radiologists to visualize the patient and exam conditions. Throughout deployment, it became clear that patient photographs opened communication between hospital staff by proxy where the proxy was the photograph. Technologists expressed that they appreciated that radiologists could see what they see, e.g., patient condition that prevented optimal radiography. Radiologists appreciated that many times their uncertainties could be resolved by the presence of the patient photograph, increasing overall interpretation confidence while decreasing calls to the floor [1, 2]. The potential of photographs to efficiently relay exam conditions between stakeholders is shown in Fig. 8.

At a subsequent deployment at another hospital, the first step after getting buy-in from the radiologists was meeting with the technologist leadership and addressing their concerns. Following this a number of training sessions were held so that all technologists could be exposed to the benefits of the technology and the minimal impact on their workflow; again, their concerns and questions were addressed.

Subsequent to the implementation, we are in periodic communication with the radiologist, technologist, quality improvement, and hospital groups to obtain their feedback directly and through surveys to continually refine the system.

#### Hospital Workflow

Throughout the deployment with the aid of patient photographs, misidentified studies were detected and reconciled. For those detected by the technologists, reconciliation could be carried out by the technologists themselves. This not only saved radiologists time and effort, but importantly enabled technologists to readily correct an unintended oversight at the source. Moreover, in at least one instance, the patient photograph allowed the technologists to further identify the correct patient to whom the initially erroneously assigned radiograph belonged. According to the technologist involved with correcting this wrong-patient error: “Without [this technology], we would not have been able to properly assign the images to the patient’s file.” In all cases, reconciliation was made easier by the presence of the photographs.

To understand the effect of patient photographs on radiologist interpretation, a preliminary study was conducted to assess radiologist accuracy and confidence in assessing lines and tubes on portable radiographs [3]. This study suggested that patient photographs increase both accuracy and confidence in radiologists’ interpretation for these studies.

#### System Support

After identifying technologists who strongly support the deployment as super users and providing training regarding the components of the programmable camera and its operation, they are now able to perform routine maintenance tasks. As a result, the frequency of visits by the camera vendor to the hospital has diminished greatly from weekly visits to monthly visits, saving time and effort while decreasing downtime.

## Discussion

Our experience in deploying an innovation in imaging informatics—a system for automated acquisition of point-of-care photographs—offered us several lessons in change management critical in deployment of any new technology clinically.

### Lessons Learned

1. *Communication and buy-in is critical*

The key component of a successful health informatics implementation in addition to hardware and software is peopleware [12]. The technology was initially devised to be entirely automated requiring no technologist interaction. However, it became clear that despite this initial guiding principle, buy-in from all stakeholders is key, and deployment is a group effort. An important lesson we learned is that communication is key. No deployment is without a change to workflow, even if initially envisioned as an automated system. All users need to know what to expect and what is expected of them. In addition, not only is users’ input on the deployment of the technology critical, but they should feel heard and valued. A thorough survey [13] of all stakeholders and in-depth training, even for automated systems, goes a long way in minimizing disruptions.

2. *Usage patterns and the end-users can change for certain benefits after deployment*

The system was initially devised for use by radiologists to detect wrong-patient errors. Wrong-patient errors are a serious underrecognized issue in radiology and prompted our implementation of the point-of-care photodocumentation technology. For example, the Pennsylvania Patient Safety Authority noted that in just 1 year (2009) in the State of Pennsylvania alone, 196 wrong-patient errors in radiology resulted in serious harm to patients [14]. This number increased to 262 by the year 2017, indicating that other solutions, such as the Joint Commission’s dual-identifier technique, are not effective by themselves. Other institutions, e.g., Memorial Sloan-Kettering Cancer Center [15] and Mayo Clinic in Rochester [16], have noted near-miss wrong-patient errors in radiology. Finally, at Emory University, a retrospective study revealed 67 near-miss wrong patient errors over a 3.5-year period [17]. However, after deployment, we found that the technologists and not radiologists were using it for detecting wrong-patient errors, since they are more responsible for correcting wrong-patient errors after detection. In addition, we unexpectedly found that wrong-side errors could be detected by this technology [3].

3. *User reactions to systems can change with time, and patience is key*

Initially, several technologists were leery of the technology and concerned that the cameras were being used to spy on their work. Eventually, technologists began to recognize that the photographs could in fact alert radiologists to patient conditions and help explain the use of non-standard (best possible) patient positioning. In addition, it is incumbent on those deploying new technologies to repeatedly survey users and continually refine the technologies.

Prior to deployment, we were given the strong message that involving humans in the loop of photograph acquisition, i.e., adding one more step to technologists’ workflow, would be detrimental to the system’s operation. However, after implementation, it became clear that technologists preferred some control over the system. This was largely driven by the need to not acquire photographs of patients who were not draped adequately for clinical reasons. Future implementations will consider study information fields to determine if a picture should be sent to PACS [18] and potentially blurring algorithms that can automatically blur sensitive regions. Thus, we implemented an override button to prevent acquisition of photographs when the technologists deemed such acquisition was inappropriate. This was an adequate compromise in that technologists did not have to routinely include a step in their workflow for every acquisition; however, with the new override button, they felt empowered to use their best judgment when needed. But every institution should develop its own guidelines based on local sensitivities [19]; note that these problems are not entirely novel and other medical specialties have tacked these situations previously—e.g., dermatologists have been using whole-body photography to document or screen for skin cancers for many years [20, 21]. Some of the technologists’ privacy concerns were addressed by fully describing the results of our prior survey of 498 patient families at a large pediatric academic hospital, which showed that while families had some privacy concerns, 97% and 96% of them were supportive of the technology if it prevented wrong-patient errors or if it could improve radiologist’s imaging interpretation [22].

4. *Expect to learn as you go*

Some of the improvements could not have been made prior to deployment. There must be a balance between being expedient and thorough. Eventually, a new system must be deployed to fully understand its impact clinically. It is not possible to wait for every concern to be addressed before deployment. However, being agile and responsive to feedback allows for concerns and problems to be addressed throughout deployment and clinical usage.

5. *Unforeseen benefits*

Another added benefit relayed by technologists was pre-exam planning, e.g., recalling specifics about a given patient in addition to bringing along an extra technologist to assist with larger patients. This benefit goes beyond just radiography. In one case, an MRI was planned for a large patient, and a photograph from a previous radiography exam was used to determine that the patient was too large for the MRI bore. This saved the patient and hospital staff both time and effort for what would have been a futile attempt.

## Conclusion

We deployed an automated point-of-care system for simultaneous acquisition of patient photographs along with portable radiographs. Initial deployment resulted in several hardware, software, and cultural challenges that needed to be thoughtfully addressed, and at this time, the system has successfully been in clinical operation for more than 3 years. We hope that our experiences and approaches in addressing the technological and cultural challenges are useful to radiology departments, healthcare informatics teams, and small businesses in the radiology space in deploying and iterating new technologies.
